# Editorial: Value-added bioproducts development through sustainable conversion and production routes

**DOI:** 10.3389/fchem.2023.1238701

**Published:** 2023-06-20

**Authors:** Hao Wang, Yulin Hu, Amarjeet Bassi, Heng Zhang

**Affiliations:** ^1^ National Key Laboratory of Green Pesticide, Key Laboratory of Green Pesticide and Agricultural Bioengineering, Ministry of Education, State-Local Joint Laboratory for Comprehensive Utilization of Biomass, Center for R&D of Fine Chemicals of Guizhou University, Guiyang, China; ^2^ Faculty of Sustainable Design Engineering, University of Prince Edward Island, Charlottetown, PE, Canada; ^3^ Department of Chemical and Biochemical Engineering, Western University, London, ON, Canada

**Keywords:** biowaste valorization, reaction mechanisms, kinetics, biofuels, biomaterials, catalysts

To respond to the need for shifting from a petroleum-based economy to a bioeconomy, it is essential to search for renewable and sustainable sources to produce fuels, chemicals, and materials. Biowaste could be an abundant and alternative resource to produce a range of fuels, chemicals, and materials (Zhang et al., 2019a; Zhang et al., 2019b; Zhang et al., 2022). Biomass is deemed a luxuriant, natural, sustainable, and renewable energy source, serving as the feedstock for the sustainable transformation of value-added bioproducts (Chen et al., 2023; Jiang et al., 2023). It is of great significance to develop biomass conversion and production approaches.

This Research Topic “*Value-added Bioproducts Development Through Sustainable Conversion and Production Routes*” explores the latest research advances in currently developing technologies, with particular attention to the underlying reaction mechanisms and kinetics, which have proven to be the most important factor in achieving process scale-up due to the wide variety of biomasses and different conversion methods. Four eminent research groups in the fields of materials chemistry and biomass resourcing gladly accepted our invitation to participate in the discussion of this Research Topic, as briefly described below:

In the paper entitled “*Synthesis of strong magnetic response ZIF-67 for rapid adsorption of Cu* ^*2+*^,” Lei et al. investigated the adsorption performance of Fe_3_O_4_@ZIF-67 catalyst for low-concentration Cu^2+^. The preparation, advantages and disadvantages, reaction conditions, and unique properties of Fe_3_O_4_@ZIF-67 catalysts, such as high chemical stability, specific surface area, adsorption, and magnetic recovery were mainly introduced. Meanwhile, exploring the effect of Fe_3_O_4_@ZIF-67 prepared in different solvents on the adsorption of Cu^2+^, and its reusability were also determined.

In the paper entitled “*Gas-solid fluidization modification of calcium carbonate for high-performance poly (butylene adipate-co-terephthalate) (PBAT) composites*,” Shang et al. focused on modifying biodegradable polypropylene dioxide (PBAT) plastics by using inorganic fillers. Experimental results showed that the gas-solid fluidization method combined the excellent modification effect of traditional wet methods with the scalability of traditional dry methods to successfully prepare biodegradable PBAT/CaCO_3_ composite materials. Meanwhile, the impact of different CaCO_3_ fillings on PBAT/CaCO_3_ composite material crystallization, mechanical performance, and flow performance was explored, laying the foundation for the large-scale preparation and application of high-performance biodegradable composite materials.

In the paper entitled “*Analysis on the thermal decomposition kinetics and storage period of biomass-based lycorine galanthamine*,” Qin et al. studied the thermal degradation behavior and mechanism of galanthamine, including its quality control, formulation process, thermal stability assessment, and production validity. Accordingly, the storage period of galanthamine hydrobromide was found to be 4–5 years at room temperature at 298.15 K. The three-dimensional dispersion mechanism for controlling the calorie-sensitive degradation of galanthamine hydrobromide was proposed following the Jander equation, random nucleation, and subsequent growth control, corresponding to the Mample unidirectional rule and the Avrami-Erofeev equation, which would provide basis for quality control and evaluation of galanthamine containing drugs.

In the paper entitled “*Insights to improve the activity of glycosyl phosphorylases from Ruminococcus albus 8 with cello-oligosaccharides*,” Storani et al. described that the structural and functional characteristics of two members of the family GH94 (CDP and CBP) from R. albus 8. Fiber dioxide phosphor phosphatase (RalCBP) and Fiber-fibrous phosphatide (RALCDP) have been identified. The latter was further analyzed by fusion of the CBM (RalCDP-CBM37) to build the intersectional mutant (RALN63CDP). RalCBP had a typical high activity for fiber dioxide. On the contrary, RalCDP extended its activity to longer-soluble or insoluble fibers with low polysaccharides. However, RalCDP produced low-fibrin polysaccharide mixtures (from fibrin triglycerides to longer low-polysaccharide), and impaired phosphosphate activity led to RalN63CDP. On the other hand, RalCDP-CBM37 enhanced the activity of polysaccharides, thus enabling the prospect of producing sugars-1P from highly available cellulosic substrates or synthesizing long-chain oligosaccharides.
